# Utility of the neonatal and pediatric sequential organ failure assessment scores in critically ill term neonates

**DOI:** 10.3389/fped.2025.1546408

**Published:** 2025-04-29

**Authors:** Lara Nicolas, James L. Wynn, Diomel de la Cruz

**Affiliations:** Department of Pediatrics, College of Medicine, University of Florida, Gainesville, FL, United States

**Keywords:** nSOFA, pSOFA, NICU, PICU, organ dysfunction, pediatric cardiac ICU, term neonate

## Abstract

**Background:**

The pediatric sequential organ failure assessment (pSOFA) and neonatal SOFA (nSOFA) scores are used to assess organ dysfunction and predict mortality in critically ill children and neonates. However, their utility in predicting mortality in term neonates admitted to pediatric ICU/pediatric cardiac ICU (PICU/PCICU) and neonatal ICU (NICU) remains unknown.

**Methods:**

We conducted a single-center retrospective cohort study of electronic health records of 4,403 and 379 term neonates admitted to NICU and PICU/PCICU, respectively. Hourly pSOFA and nSOFA scores were calculated. The primary outcome was in-hospital mortality. The area under the receiving operating characteristic curve (AUROC) for mortality was calculated.

**Results:**

Both scores predicted mortality in both settings (AUROC range, 0.79–0.95). The pSOFA showed a larger difference between survivors and non-survivors in the PICU/PCICU cohort, while nSOFA captured critical mortality risk factors in neonates across both settings.

**Conclusions:**

Both pSOFA and nSOFA predicted mortality with good to very good discrimination in critically ill term neonates admitted to PICU/PCICU and NICU settings.

## Introduction

The sequential organ failure assessment (SOFA) score emerged as a valuable tool to quantify the extent of organ dysfunction in six organ systems (i.e., respiratory, coagulation, hepatic, cardiovascular, renal, and neurologic) and to predict adverse outcomes, such as mortality and the need for intensive care unit (ICU) admission. Early identification and accurate assessment of organ dysfunction in critically ill infants are crucial for timely intervention and improved outcomes. Initially developed for adults, the SOFA score has been adapted and validated for pediatric (pSOFA) and neonatal (nSOFA) populations to address the unique physiological characteristics and clinical presentations in these age groups (1–8). The pSOFA is frequently used in the pediatric intensive care unit (PICU) and pediatric cardiac ICU (PCICU) populations, while the nSOFA is used in the neonatal ICU (NICU) setting (Supplementary Tables S1, S2).

The pSOFA and nSOFA are valuable tools to quantify organ dysfunction and predict adverse outcomes, including mortality, in critically ill children and neonates. The pSOFA predicts mortality risk in pediatric emergency departments and intensive care units for patients with sepsis (1, 2, 5, 6). Similarly, the nSOFA has demonstrated excellent utility for mortality in neonates, especially those born prematurely or with extremely low birth weight (<1,000 g), and across various specific neonatal conditions, including sepsis, necrotizing enterocolitis, and congenital heart disease (3, 4, 9–14).

Critically ill term neonates may be cared for in the NICU or PICU. However, there has not been a direct comparison of the utility of the pSOFA and nSOFA to predict mortality in the term neonate cared for in these distinct ICU settings. We aimed to measure and compare the utility of the pSOFA and nSOFA for in-hospital mortality among critically ill term neonates admitted to the neonatal and pediatric ICU.

## Methods

### Patients

We conducted a single-center, retrospective, observational cohort study approved by the University of Florida (UF) Institutional Review Board prior to data collection. This retrospective study was deemed exempt from patient consent by the UF Institutional Review Board as it posed minimal risk to participants. An integrated data repository was created with all clinical data in the electronic health record (EHR) for all patients admitted to the UF Health NICU (1/1/2012 and 4/1/2023), PICU (1/1/2012–7/1/2020), and PCICU (1/01/2014–7/1/2020). Eligible patients are those who completed at least 37 weeks' gestation and were ≤30 days old when admitted. There were no exclusion criteria. Patients were classified as NICU or PICU/PCICU based on the first location of admission. Thus, the cohorts were mutually exclusive. Each cohort was assessed using both metrics (nSOFA and pSOFA). Mortality was defined as in-hospital mortality.

### pSOFA and nSOFA

The pSOFA score includes the assessment of six organ systems: respiratory (PaO_2_/FiO_2_ ratio or the need for mechanical ventilation), cardiovascular (blood pressure and vasoactive medications), hepatic (bilirubin levels), coagulation (platelet count), renal (serum creatinine or urine output), and neurologic [Glasgow Coma Scale (GCS) score or the pediatric GCS equivalent] (1). The nSOFA evaluates three systems: respiratory (FiO_2_, SpO_2_, and intubated status), cardiovascular (systemic steroids, qualitative vasoactive–inotropic drug exposure), and hematologic (platelet count) (3). Because the pediatric GCS is not recorded for NICU patients, the pSOFA neurologic subscore could not be calculated in the NICU cohort. Thus, the pSOFA scores among NICU patients were the sum of the respiratory, cardiovascular, hepatic, renal, and coagulation subscores only. Hourly pSOFA or nSOFA scores were calculated using the newest values available, thus reflecting the pSOFA and nSOFA scores that would have been derived on assessment by a bedside clinician at that time. Laboratory measurements were performed at the discretion of the clinical team and were not hourly. To permit hourly calculations for pSOFA and nSOFA subscores based on laboratory values [hematologic/coagulation (platelet), renal (creatinine), and hepatic (bilirubin)], a last-one-copied-forward approach was used until a new value was available. When calculation of more than one nSOFA score was possible within a single hour for a single patient, the maximum nSOFA score based on simultaneously calculated nSOFA components was used.

### Statistical analysis

The non-parametric continuous variables were summarized as medians with quartiles and compared between groups using the Mann–Whitney test. The categorical variables were presented as percentages and compared using the Chi-square test. The threshold for statistical significance was <0.05 for two-sided tests. The area under the receiver operating characteristic curves (AUROCs) for mortality based on the nSOFA or pSOFA score at described time points were calculated using the Wilson/Brown method. Analyses were performed using GraphPad Prism (version 10.3; group comparisons and graph productions) and R (version 4.2.2; calculation of hourly nSOFA/pSOFA from raw EHR data).

## Results

### Patients

We identified 4,403 and 379 term neonates admitted to the neonatal intensive care unit (NICU) and pediatric intensive care unit (PICU)/pediatric cardiac intensive care unit (PCICU), respectively (Table 1). Term neonates admitted to the PICU/PCICU were older and had a longer length of stay than infants admitted to the NICU [11 days (IQR: 5, 20) vs 0 days (IQR: 0, 0), *p* < 0.0001, and 7 days (IQR: 4, 21) vs 6 days (IQR: 4, 13), *p* = 0.02, respectively). Compared to term neonates in the NICU, term neonates admitted to the PICU/PCICU were significantly more likely to receive mechanical ventilation (39% vs. 23%; *p* < 0.0001), vasoactive–inotropic medications (30% vs. 12%; *p* < 0.0001), sedative infusions (36% vs. 19%; *p* < 0.0001), and prostaglandin infusion (20% vs. 13%; *p* = 0.0005). No significant differences were seen between populations in terms of birth weight, proportion of male infants, rate of major congenital anomalies, proportion of infants who underwent surgery, or mortality.

**Table 1:** Cohort demographics.

Variable	NICU (*n* = 4,403)	PICU/PCICU (*n* = 379)	*p*-value
Age at admission (days) median (interquartile range; IQR)	0 (0, 0)	11 (5, 20)	<0.0001 mw
Admission weight (grams) median (interquartile range; IQR)	3,260 (2,864, 3,642)	3,289 (2,892, 3,714)	0.22 mw
Race/ethnicity, *n* (%)^a^			0.41 chi
Black	948 (22)	79 (21)	
White	2,692 (61)	239 (63)	
Other^b^	763 (17)	61 (16)	
Hispanic	416 (9)	25 (7)	
Male, *n* (%)	2,482 (56)	224 (59)	0.30 chi
Received mechanical ventilation, *n* (%)	1,015 (23)	148 (39)	<0.0001 chi
Received vasoactive–inotropic medications, *n* (%)	515 (12)	114 (30)	<0.0001 chi
Received prostaglandin infusion, *n* (%)	586 (13)	75 (20)	0.0005 chi
ECMO in ICU, *n* (%)	101 (2.3)	14 (3.7)	0.09 chi
Received sedative infusion, *n* (%)	819 (19)	135 (36)	<0.0001 chi
Major congenital anomaly, *n* (%)	893 (20)	83 (22)	0.45 chi
Operation during hospitalization, *n* (%)	726 (16)	55 (15)	0.32 chi
Length of stay (days), median (IQR)	6 (4, 13)	7 (4, 21)	0.02 mw
28-day mortality, *n* (%)	58 (1)	8 (2)	0.20 chi
Death, *n* (%)	82 (2)	12 (3)	0.09 chi
Age at death (days), median (IQR)	11 (4, 31)	18 (10, 88)	0.11 mw

chi, Chi-square; mw, Mann–Whitney.

^a^ Family's response to question of race/ethnicity.

^b^ Asian [*n* = 103 (96 NICU + 7 PICU_PCICU)], American Indian (*n* = 11 NICU), multi-race [*n* = 120 (114 NICU + 6 PICU_PCICU)], Pacific Islander (*n* = 1 NICU), unknown or other [*n* = 589 (106 NICU + 435 PICU_PCICU; 31 NICU + 17 PICU_PCICU)].

## Predictive performance of nSOFA and pSOFA for mortality

In the NICU cohort, both nSOFA and pSOFA demonstrated good to very good discrimination for 28 days and overall mortality with AUROCs ranging from 0.84 to 0.88 within the first 72 h (Table 2). Both scores for the PICU/PCICU cohort also showed good to very good discriminatory ability, with AUROCs ranging from 0.84 to 0.90. pSOFA exhibited higher AUROCs compared with nSOFA in the PICU/PCICU group. When considering maximum scores over longer durations, including 7 days, 28 days, or the entire encounter, both scores maintained excellent discrimination for mortality in the NICU cohort, with AUROCs consistently above 0.89. The discriminatory accuracy in the PICU/PCICU cohort decreased slightly over time, particularly for nSOFA, with AUROCs ranging from 0.79 to 0.85 for 28-day and encounter mortality, respectively.

**Table 2:** Area under receiver operating characteristic curve for mortality by maximum score type, timing, and cohort.

Time after admission	24 h		48 h		72 h	
	AUROC (CI)	*p*-value	AUROC (CI)	*p*-value	AUROC (CI)	*p*-value
nSOFA						
NICU	0.84 (0.79, 0.89)	<0.0001	0.87 (0.82, 0.92)	<0.0001	0.87 (0.82, 0.92)	<0.0001
PICU/PCICU	0.84 (0.71, 0.97)	<0.0001	0.87 (0.77, 0.97)	<0.0001	0.85 (0.75, 0.95)	<0.0001
pSOFA						
NICU	0.86 (0.82, 0.90)	<0.0001	0.88 (0.84, 0.92)	<0.0001	0.88 (0.84, 0.92)	<0.0001
PICU/PCICU	0.89 (0.81, 0.98)	<0.0001	0.90 (0.81, 0.99)	<0.0001	0.88 (0.79, 0.98)	<0.0001
	7 days		28 days		Encounter	
	AUROC (CI)	*p*-value	AUROC (CI)	*p*-value	AUROC (CI)	*p*-value
nSOFA						
NICU	0.90 (0.85, 0.94)	<0.0001	0.92 (0.89, 0.95)	<0.0001	0.95 (0.94, 0.96)	<0.0001
PICU/PCICU	0.81 (0.71, 0.91)	0.0003	0.79 (0.73, 0.85)	0.0006	0.85 (0.77, 0.92)	<0.0001
pSOFA						
NICU	0.89 (0.86, 0.93)	<0.0001	0.91 (0.87, 0.94)	<0.0001	0.93 (0.90, 0.96)	<0.0001
PICU/PCICU	0.86 (0.76, 0.96)	<0.0001	0.87 (0.82, 0.93)	<0.0001	0.92 (0.87, 0.97)	<0.0001

## Discussion

This study represents the first direct comparison of the pSOFA and nSOFA to predict mortality in critically ill term neonates admitted to pediatric and neonatal ICUs (1–4, 9, 13, 15). These results emphasize the clinical utility of both nSOFA and pSOFA scores for mortality risk stratification in critically ill children. The pSOFA showed greater area under the receiving operating characteristic curve (AUROC) for mortality in PICU/PCICU and in the first few hours after admission in the NICU population. The nSOFA showed greater AUROC over 28 days and the encounter in the NICU population. These findings may lead to improved care for shared neonatal patient populations in neonatal and pediatric settings.

As anticipated, our study revealed distinct differences in neonatal patient populations cared for in these pediatric ICU and neonatal ICU settings. The lower number of patients but higher prevalence of prostaglandin E1 (PGE1) use, mechanical ventilation, and vasoactive–inotropic support in the PICU/PCICU cohort aligns with the observed increases in shock and respiratory failure. Additionally, the relatively low mortality rate in the PICU/PCICU population, while clinically positive, may limit the analysis of organ dysfunction's specific contribution to mortality risk due to the diverse paths to death in this cohort.

Despite baseline cohort differences, both scoring systems demonstrated robust predictive capabilities for mortality, with consistently high AUROC values across both cohorts. The AUROCs for the pSOFA and nSOFA were comparable in the NICU cohort, suggesting the three organ system-based nSOFA effectively captures the critical components that underpin neonatal mortality risk in the NICU. The pSOFA often exhibited higher AUROC values, particularly in the PICU/PCICU cohort, indicating potential superiority in predicting mortality for older neonates (Table 2). This finding may be attributed to the broader measure of organ dysfunction in the pSOFA, including assessment of hepatic, renal, and central nervous systems, which are not currently measured in the nSOFA score. The pSOFA is more likely to be familiar to PICU and PCICU staff, and the nSOFA is more likely to be familiar to NICU staff. We recommend using the score available, but know that if you have to use the other, both are effective in the neonatal population.

### Limitations

This study has limitations inherent to all single-center, retrospective analyses. We acknowledge that retrospective data cannot guide clinical practice. Pediatric GCS scores are not routinely collected for term neonates cared for in the NICU. This limitation required us to examine the utility of an altered pSOFA score in the NICU cohort. A limited number of deaths (*n* = 12) among neonates cared for in the PICU/PCICU cohort may affect the generalizability of our findings at other institutions. However, this study included nearly 5,000 term infants cared for over a decade in three ICU settings where both hourly nSOFA and pSOFA scores were calculated throughout the entire hospital stay. We chose to use the maximum score for our metrics. We recognize incorporation of repeated measures in the future may improve the utility of these scores for mortality risk prediction in this population.

## Conclusion

Among critically ill term neonates admitted to pediatric intensive care and neonatal intensive care settings, both pSOFA and nSOFA showed utility in predicting mortality, with non-survivors consistently exhibiting higher scores.

These findings warrant further investigation into the distinct pathophysiological processes underlying organ dysfunction and mortality in different pediatric populations.

## Data Availability

The raw data supporting the conclusions of this article will be made available by the authors, without undue reservation.
