# Supplementary material for: Utility of the neonatal and pediatric sequential organ failure assessment scores in critically ill term neonates
Source: Front Pediatr. 2025 Apr 29;13:1546408. doi: 10.3389/fped.2025.1546408 (PMC12069352; doi:10.3389/fped.2025.1546408)
Supplement: Supplementary file 1 [file Supplementaryfile1.docx]

**Table 1. Cohort demographics**

| Variable | NICU (n=4403) | PICU/PCICU (n=379) | p-value |
| --- | --- | --- | --- |
| Age at admission (days) median (interquartile range; IQR) | 0 (0, 0) | 11 (5, 20) | <0.0001 mw |
| Admission weight (grams) median (interquartile range; IQR) | 3260 (2864, 3642) | 3289 (2892, 3714) | 0.22 mw |
| Race/ethnicity, n (%)* |  |  | 0.41 chi |
| Black | 948 (22) | 79 (21) |  |
| White | 2692 (61) | 239 (63) |  |
| Other** | 763 (17) | 61 (16) |  |
| Hispanic | 416 (9) | 25 (7) |  |
| Male, n (%) | 2482 (56) | 224 (59) | 0.30 chi |
| Received mechanical ventilation, n (%) | 1015 (23) | 148 (39) | <0.0001 chi |
| Received vasoactive-inotropic medications, n (%) | 515 (12) | 114 (30) | <0.0001 chi |
| Received prostaglandin infusion, n (%) | 586 (13) | 75 (20) | 0.0005 chi |
| ECMO in ICU, n (%) | 101 (2.3) | 14 (3.7) | 0.09 chi |
| Received sedative infusion, n (%) | 819 (19) | 135 (36) | <0.0001 chi |
| Major congenital anomaly, n (%) | 893 (20) | 83 (22) | 0.45 chi |
| Operation during hospitalization, n (%) | 726 (16) | 55 (15) | 0.32 chi |
| Length of stay (days), median (IQR) | 6 (4, 13) | 7 (4, 21) | 0.02 mw |
| 28-day mortality, n (%) | 58 (1) | 8 (2) | 0.20 chi |
| Death, n (%) | 82 (2) | 12 (3) | 0.09 chi |
| Age at death (days), median (IQR) | 11 (4, 31) | 18 (10, 88) | 0.11 mw |

** Family’s response to question of race/ethnicity*

*** Asian (n=103 (96 NICU + 7 PICU_PCICU)), American Indian (n=11 NICU), Multi-race (n= 120 (114 NICU + 6 PICU_PCICU)), Pacific Islander (n=1 NICU), Unknown or Other (n=589 (106 NICU + 435 PICU_PCICU; 31 NICU+17 PICU_PCICU)).*
