# Supplementary material for: Utility of the neonatal and pediatric sequential organ failure assessment scores in critically ill term neonates
Source: Front Pediatr. 2025 Apr 29;13:1546408. doi: 10.3389/fped.2025.1546408 (PMC12069352; doi:10.3389/fped.2025.1546408)
Supplement: Supplementary file 2 [file Supplementaryfile2.docx]

**Table 2. Area under receiver operating characteristics curve for mortality by maximum score type, timing, and cohort**

|  | 24 hours | | 48 hours | | 72 hours | |
| --- | --- | --- | --- | --- | --- | --- |
|  | AUROC (CI) | p-value | AUROC (CI) | p-value | AUROC (CI) | p-value |
| nSOFA |  |  |  |  |  |  |
| NICU | 0.84 (0.79, 0.89) | <0.0001 | 0.87 (0.82, 0.92) | <0.0001 | 0.87 (0.82, 0.92) | <0.0001 |
| PICU/PCICU | 0.84 (0.71, 0.97) | <0.0001 | 0.87 (0.77, 0.97) | <0.0001 | 0.85 (0.75, 0.95) | <0.0001 |
| pSOFA |  |  |  |  |  |  |
| NICU | 0.86 (0.82, 0.90) | <0.0001 | 0.88 (0.84, 0.92) | <0.0001 | 0.88 (0.84, 0.92) | <0.0001 |
| PICU/PCICU | 0.89 (0.81, 0.98) | <0.0001 | 0.90 (0.81, 0.99) | <0.0001 | 0.88 (0.79, 0.98) | <0.0001 |
|  | 7 days | | 28 days | | Encounter | |
|  | AUROC (CI) | p-value | AUROC (CI) | p-value | AUROC (CI) | p-value |
| nSOFA |  |  |  |  |  |  |
| NICU | 0.90 (0.85, 0.94) | <0.0001 | 0.92 (0.89, 0.95) | <0.0001 | 0.95 (0.94, 0.96) | <0.0001 |
| PICU/PCICU | 0.81 (0.71, 0.91) | 0.0003 | 0.79 (0.73, 0.85) | 0.0006 | 0.85 (0.77, 0.92) | <0.0001 |
| pSOFA |  |  |  |  |  |  |
| NICU | 0.89 (0.86, 0.93) | <0.0001 | 0.91 (0.87, 0.94) | <0.0001 | 0.93 (0.90, 0.96) | <0.0001 |
| PICU/PCICU | 0.86 (0.76, 0.96) | <0.0001 | 0.87 (0.82, 0.93) | <0.0001 | 0.92 (0.87, 0.97) | <0.0001 |
